# Distinct Gut Microbiota and Arachidonic Acid Metabolism in Obesity-Prone and Obesity-Resistant Mice with a High-Fat Diet

**DOI:** 10.3390/nu16111579

**Published:** 2024-05-23

**Authors:** Huixia Zhang, Shiqi Chen, Liu Yang, Shuai Zhang, Linqian Qin, Haiyang Jiang

**Affiliations:** National Key Laboratory of Veterinary Public Health and Safety, Department of Veterinary Pharmacology and Toxicology, College of Veterinary Medicine, China Agricultural University, Beijing 100193, China; zhanghuixia2008@163.com (H.Z.); chenshiqi03@gmail.com (S.C.); y_13121376788@163.com (L.Y.); zsxxsc@163.com (S.Z.); qinlqa@126.com (L.Q.)

**Keywords:** obesity prone, obesity resistant, gut microbiota, widely targeted metabolomics, arachidonic acid metabolism

## Abstract

An imbalance of energy intake and expenditure is commonly considered as the fundamental cause of obesity. However, individual variations in susceptibility to obesity do indeed exist in both humans and animals, even among those with the same living environments and dietary intakes. To further explore the potential influencing factors of these individual variations, male C57BL/6J mice were used for the development of obesity-prone and obesity-resistant mice models and were fed high-fat diets for 16 weeks. Compared to the obesity-prone mice, the obesity-resistant group showed a lower body weight, liver weight, adipose accumulation and pro-inflammatory cytokine levels. 16S rRNA sequencing, which was conducted for fecal microbiota analysis, found that the fecal microbiome’s structural composition and biodiversity had changed in the two groups. The genera *Allobaculumbiota*, *SMB53*, *Desulfovibrio* and *Clostridium* increased in the obesity-prone mice, and the genera *Streptococcus*, *Odoribacter* and *Leuconostoc* were enriched in the obesity-resistant mice. Using widely targeted metabolomics analysis, 166 differential metabolites were found, especially those products involved in arachidonic acid (AA) metabolism, which were significantly reduced in the obesity-resistant mice. Moreover, KEGG pathway analysis exhibited that AA metabolism was the most enriched pathway. Significantly altered bacteria and obesity-related parameters, as well as AA metabolites, exhibited strong correlations. Overall, the phenotypes of the obesity-prone and obesity-resistant mice were linked to gut microbiota and AA metabolism, providing new insight for developing an in-depth understanding of the driving force of obesity resistance and a scientific reference for the targeted prevention and treatment of obesity.

## 1. Introduction

Obesity has become one of the most primary public health problems worldwide since it can disturb the state of homeostasis and lead to dysfunction of the immune system and chronic inflammation [1,2]. Obesity is also a risk factor for metabolic diseases, cancers and others [3,4]. The occurrence of obesity is caused by various factors, such as genetic or epigenetic effects, dietary habits and individual behaviors of energy expenditure [5,6], among which the imbalance between energy intake and expenditure is commonly considered the underlying reason for obesity [7]. However, individual variations in susceptibility to obesity indeed exist among humans and animals, e.g., among those within the same living environments and with the same dietary intakes, body weights and fat masses substantially differ. In this context, it would be substantially helpful to figure out the underlying causes of these variations for obesity prevention and therapy.

The gut microbiota is now considered an organ that is strongly correlated with the development of obesity, which has gained significant research interest [8,9,10]. The exploration of the gut microbiota is important not only for obesity prevention and treatment but also for the long-term maintenance of obesity therapy [11,12]. Diets largely affect the composition and diversity of the gut microbiota [13], such as high-fat diets, which are generally considered to cause trends in obesity. Accordingly, individuals that are sensitive to a high-fat diet with significant body weight gain are defined as the obesity-prone group (HFD-P), and those who still remain lean despite having the same or a higher-fat diet are defined as the obesity-resistant group (HFD-R). The gut microbiota of obesity-resistant individuals exhibit an increased taxonomic diversity compared to that of obesity-prone individuals [14], suggesting that the microbiota of humans with obesity are relatively simple. Also, lean animals showed an enriched presence of the *Bacteroidetes* phylum and a diminished proportion of the *Firmicutes* phylum in gut microbiota [15,16,17], while Schwiertz et al. reported that the ratio of *Firmicutes* to *Bacteroidetes* was reduced in humans who were overweight or obese [18]. In addition, the increase in *Parasutterella*, from the *Proteobacteria* phylum, in obesity-prone mice is highly linked to the development of obesity [19], and obesity-resistant rats had significantly higher relative abundances of the genus *Clostridium*, from the *Firmicutes* phylum, and the genus *Akkermansia*, from the *Verrucomicrobia* phylum [20]. Moreover, humans with obesity had higher abundances of *Verrucomicrobia*, *Acidaminococcus*, *Lachnospira* and *Saccharibacteira* and lower abundances of *Lentisphaerae* [21]. Additionally, a previous publication stated that humans with overweight or obesity had a higher abundance of *Akkermansia muciniphila*, which was correlated with a healthier metabolic status [22]. All of these studies indicate that the composition of intestinal flora is bound up with obese/lean; however, the precise bacteria that manipulate the phenotypes still remain unclear.

It is well known that obese and lean individuals have quite different statuses related to metabolism, and the susceptibilities to obesity considerably differ among individuals. In this context, researchers are particularly interested in the investigation of metabolic differences between obesity-prone and obesity-resistant individuals to further explore their intrinsic mechanisms of metabolic regulation. Wei et al. reported that the compositions of bile acids (BAs) were altered, especially the non-12-OH BAs (ursodeoxycholate, chenodeoxycholate, and lithocholate), which were significantly reduced in subjects with obesity and obesity-prone mice [23]. Moreover, obesity-resistant mice showed diminished short-chain fatty acids (SCFAs) metabolism in feces, and the reverse effects were found in obesity-prone mice [24]. Similar results were found in humans: the mean concentration of the total SCFAs in the feces of subjects with obesity was more than 20% higher than that in lean subjects, and the highest growth was propionate followed by butyrate [20]. Furthermore, metabolomics and targeted lipidomics analyses revealed that the significantly differential metabolites were gamma-glutamyl, branched-chain amino acids and triacylglycerols between lean and obese subjects [25]. Likewise, other reports have demonstrated prominent differences in the composition of lipids and contents of fecal lipids as well [26,27]. In addition, the liver metabolic profiles of obesity-prone rats showed increases in hexadecanoic acid, tetradecanoic acid, octadecylic acid, hexadecenoic acid and several glucogenic and ketogenic amino acids and decreases in arachidonic acid and octadecadienoic acid [28]. Although these findings show metabolic differences to some extent, the exact changes in the metabolites between obesity-prone and obesity-resistant individuals are not fully understood.

The objective of the present work was to further explore the potential influencing factors of the susceptibility to obesity. To this end, high-fat diets were used to induce obesity-prone and obesity-resistant mice, and 16S rRNA sequencing and widely targeted metabolomics analysis were implemented for the investigation of differential bacteria and metabolites between the two groups. Accordingly, the correlation analyses between screened bacteria, differential metabolites, and obesity-prone/obesity-resistant phenotypes were carried out to explore the internal relationship among them. The findings of this work provide novel insights for developing an in-depth understanding of the driving force of obesity resistance, and a scientific reference for the targeted prevention and therapy of obesity.

## 2. Materials and Methods

### 2.1. Animal Experiments

A total of 40 four-week-old male C57BL/6J mice were ordered from Vital River Laboratory (Beijing, China) and were divided into four mice per cage. All mice were allowed free access to water and food under 22 °C ± 2 °C and 12 h light and dark cycles. All animal work was conducted with the approval of the Ethics Committee of China Agricultural University (AW11011202-2-15).

Prior to the investigation, all animals were acclimatized for 7 days. Subsequently, all mice were randomly divided into two groups. Eight mice in the control group received a chow-diet (H10010), and thirty-two mice in the experimental group received a high-fat-diet (H10060), purchased from Beijing HFK Bioscience Co., Ltd. (Beijing, China), for 16 weeks. The chow diet contained 10% fat, 20% protein and 70% carbohydrate (kcal), and the high-fat diet contained 60% fat, 20% protein and 20% carbohydrate (kcal). Body weights and dietary intakes of mice were recorded weekly during the whole experiment. At the end of the sixteenth week, the average body weight of the control group was 28.8 ± 1.43 g. The mice in the experimental group whose body weight was 1.2 times higher than that of the control group were taken as the obesity-prone mice, and those whose body weight was 1.1 times lower than that of the control group were taken as the obesity-resistant mice. Based on this criteria, 24 mice were taken as the obesity-prone mice, while only 5 mice were considered as the obesity-resistant mice. For the obesity-prone group, 8 mice were randomly selected for further analysis. Fresh feces samples were collected and rapidly frozen using liquid nitrogen at the end of the sixteenth week, and then stored at −80 °C until further test. Next, all mice were anesthetized by isoflurane and euthanized. Blood samples were obtained via cardiac punctures and placed at room temperature for 30 min. Afterwards, all blood samples were centrifuged at 5000 rpm for 20 min at 4 °C, and the supernatants were carefully drawn to obtain serum samples. The brown adipose tissues, white adipose tissues (epididymal adipose, subcutaneous adipose, visceral adipose and fat pad) and livers were harvested and weighed. All samples were stored at −80 °C until further analyses.

### 2.2. Determination of Biomarkers in Serum

ELISA kits for the determination of tumor necrosis factor-alpha (TNF-α), interleukin (IL)-1β, interleukin (IL)-6, and interleukin (IL)-10 in serum were purchased from Beijing Solarbio Science & Technology Co., Ltd. (Beijing, China). The content measurements of these inflammatory cytokines were performed following the instructions provided by the manufacturer.

### 2.3. Gut Microbiota Analysis

Feces samples stored at −80 °C were used for the microbiota analyses. Total genomic DNA was extracted using OMEGA Soil DNA Kit from Omega Bio-Tek (Norcross, GA, USA). To evaluate the quantity and quality of the extracted DNAs, we used a NanoDrop NC2000 spectrophotometer and agarose gel electrophoresis, respectively.

In this study, the hypervariable V3–V4 regions of 16S rRNA gene of bacteria were chosen for PCR amplification with the specific primer of 338F (5′-barcode + ACTCCTACGGGAGGCAGCA-3′) and 806R (5′-GGACTACHVGGGTWTCTAAT-3′). Different 7-bp barcodes were inserted in the front of primers to distinguish the different samples in the same library. For further analysis, purification and quantification of PCR amplification products were carried out accordingly. Subsequently, equivalent amplicons were pooled and 2 × 250 bp paired-end sequenced on the Illlumina NovaSeq platform.

Sequence analyses were carried out on QIIME2 [29] with minor modification based on the official guidance and R software packages (version 3.2.0). The DADA2 plugin [30] was used for sequences to further analyze by quality filtration, denoising, merging and removing chimera. Taxonomy was conducted using amplicon sequence variants (ASVs) by the naive Bayes taxonomy classifier with the classify-sklearn algorithm [31] against the Greengenes Rlease 13.8 Database “http://greengenes.secondgenome.com/ (accessed on 26 May 2022)” in feature-classifier plugin. Bio-diversity was assessed by a plugin.

### 2.4. Widely Targeted Metabolomics Analysis

Serum samples were thawed on ice and vortexed for 10 s to mix well. The protein precipitation method was used for sample preparation. An amount of 50 μL of serum sample was placed into a 2 mL microcentrifuge tube and spiked with 300 μL of 25% acetonitrile/75% methanol solution containing internal standards, then vortexed for 3 min to precipitate proteins. After that, all samples were centrifuged at 12,000 rpm for 10 min at 4 °C. Next, 200 μL of supernatant was taken out into a 2 mL microcentrifuge tube and placed at −20 °C for 30 min, and centrifuged at 12,000 rpm for 3 min at 4 °C. Finally, 180 μL of supernatant was transferred to a vial for LC-MS/MS analysis.

Extracted samples were analyzed by an AB Sciex LC-MS/MS system, ExionLC AD UPLC coupled with a QTRAP^®^ System MS. Chromatographic separation was achieved on a Waters T3 C18 column (2.1 mm × 100 mm, 1.8 µm). The flow rate was 0.4 mL/min. The mobile phases were water containing 0.1% formic acid (A) and acetonitrile containing 0.1% formic acid (B). The gradient program was set as 5% B at 0 min, 90% B at 11.0 min, 90% B at 12.0 min, 5% B at 12.1 min, 5% at 14.0 min, and the column temperature was maintained at 40 °C. Then, 2 μL was injected to LC-MS/MS for analysis.

All targeted transitions were monitored by multiple reaction monitoring (MRM) in positive and negative modes. The ESI source temperature was set at 500 °C. The ion spray voltage (IS) was set as 5500 V and −4500 V for positive and negative mode, respectively. The ion source gas I, gas II and curtain gas were 55, 60, and 25.0 psi, respectively. The software Analyst (1.6.3, AB Sciex, Framingham, MA, USA) was used for the control of sample injection and data processing.

### 2.5. Statistical Analysis

Graphpad Prism 9.0 (San Diego, CA, USA) was used for data plotting and statistical analysis. Unpaired *t*-test was used for statistical comparisons between two groups. Data were expressed as the mean ± SD or medians. Multivariate analysis was performed by R package (version 3.5.1). The data analysis and figures plotting of the fecal microbiota and Spearman correlation analyses were carried out by the genescloud tools “https://www.genescloud.cn (accessed on 20 February 2024)”. The *p*-value < 0.05 was considered statistically significant.

## 3. Results

### 3.1. Distinct Effects on Body Weight and Physiological Properties of Mice with High-Fat Diets

**Figure 1:**
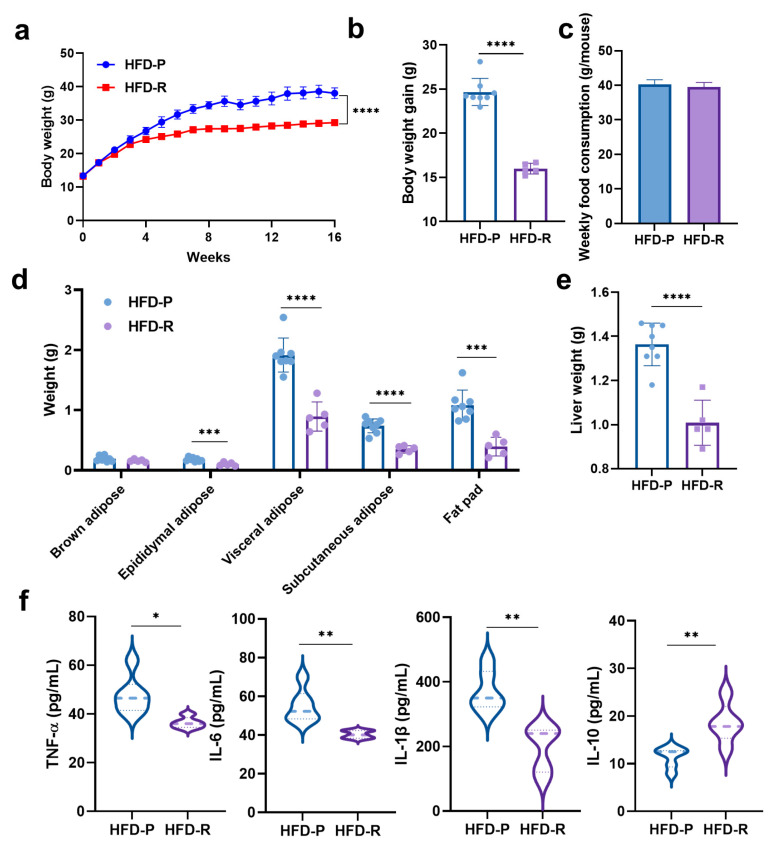
Effects on body weight and physiological properties of obesity-prone and obesity-resistant mice induced by high-fat diets (i.e., obesity-prone, HFD-P; obesity-resistant, HFD-R). (**a**) Body weight–time curves; (**b**) body weight gain; (**c**) weekly food consumption; (**d**) different adipose tissues weight; (**e**) liver weight; (**f**) levels of serum inflammatory cytokines in mice. Data are presented as means ± SD for figure (**a**–**e**) and medians for figure (**f**) (HFD-P, *n* = 8 mice/group; HFD-R, *n* = 5 mice/group). Statistical analysis used unpaired t-test. * *p* < 0.05; ** *p* < 0.01; *** *p* < 0.001; **** *p* < 0.0001.

At the very beginning, there were no notable variances in the initial body weight between the two groups. All mice were fed the same high-fat diets at the same time for 16 weeks, but the effects were quite different between HFD-P and HFD-R mice (Figure 1). Figure 1a shows the body weight–time curves of mice, and the final body weights of the HFD-P group exhibited a significant increase to 38.07 ± 1.58 g, which was approximately 1.3 times of that in the HFD-R group. The differences in body weight gains between the two groups were also statistically significant (Figure 1b). Of note, the weekly food consumption per mouse was basically the same (Figure 1c).

Excessive accumulation of body fat is a hallmark of obesity; therefore, we measured the weights of different adipose tissues (brown adipose, epididymal adipose, visceral adipose, subcutaneous adipose and fat pad) to evaluate the susceptibility to high-fat diets of mice in the two groups. As shown in Figure 1d, with the exception of brown adipose tissues, the increases in the weight of all white adipose tissues in the HFD-R group were significantly less than those in the HFD-P group. Also, the ratios of different tissue weights to body weight were calculated and are plotted in Figure S1, indicating that the body fat rate of the HFD-R group was significantly lower than that of the HFD-P group. Additionally, compared to the HFD-P mice, the HFD-R mice had a significantly lower liver weight (Figure 1e), whereas the liver weight normalized to body weight was not significantly different (Figure S1).

In addition to the body weight gain and the accumulation of adipose tissues mentioned above, systemic inflammation is also an important feature of obesity. To assess the differences in inflammation between the HFD-P group and the HFD-R group, ELISA kits were used for the quantification of inflammatory cytokines. As shown in Figure 1f, all pro-inflammatory cytokines in the HFD-R group exhibited significantly lower levels compared to the HFD-P group, while the levels of the anti-inflammation cytokine were just the opposite, which was consistent with our expectations.

### 3.2. Different Fecal Microbiota Composition between Obesity-Prone and Obesity-Resistant Mice

**Figure 2:**
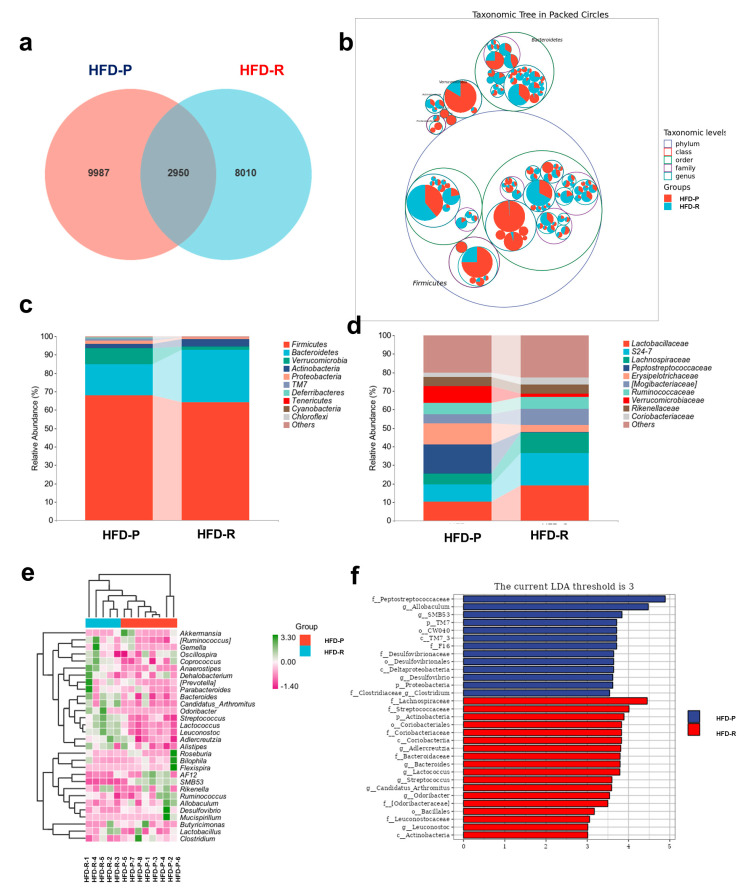
Distinct fecal microbiota composition of obesity-prone (HFD-P) and obesity-resistant (HFD-R) mice with high-fat diets. (**a**) Venn diagram; (**b**) taxonomic tree in packed circles. On the base of the tree map of fecal microbiota classification, the abundance of each ASV group was added to the map with a pie chart. The largest circle represents phylum level, and the gradually shrinking circle represents class, order, family and genus level. The innermost dot area represents the abundance and the composition proportion of ASVs in each group; (**c**) relative abundance of dominant bacteria at the phylum taxa level; (**d**) relative abundance of dominant bacteria at the family taxa level; (**e**) heatmap of top 30 bacteria at the genus taxa level; (**f**) LefSe analysis of fecal microbiota.

The 16S rRNA sequencing was carried out for the investigation of the composition alterations of the fecal microbiota between the HFD-R group and the HFD-P group (Figure 2). A Venn diagram was used to exhibit the differences in microbiota community between the two groups, for which a total of 20947 ASVs were determined with 2950 common ASVs (Figure 2a). A dendrogram of a taxonomic tree in packed circles (Figure 2b) showed that the top 100 ASVs in abundance were mainly distributed in *Firmicutes* and *Bacteroidetes*, among which ASVs in *Firmicutes* were the most abundant, and similar results at the phylum taxa level are also shown in Figure 2c. The HFD-R group had a slight decrease in *Firmicutes* and increase in *Bacteroidetes*; simultaneously, a relatively lower ratio of *Firmicutes/Bacteroidetes* (*F/B*) was observed(Figure S2), while there was no statistical difference. Additionally, the relative abundance of the main bacteria at the family taxa level showed that *Peptostreptococcaceae*, *Erysipelotrichaceae* and *Verrucomicrobiaceae* were highly diminished in the HFD-R group, whereas, *Lactobacillaceae*, *S24-7* and *Lachnospiraceae* presented opposite results (Figure 2d). A heatmap of the top 30 ASVs in relative abundance at the genus level is displayed in Figure 2e, wherein the genera *Streptococcus*, *Lactococcus* and *Leuconostoc* were increased in the HFD-R group with a decline in *AF12* and *SMB53*, compared to the HFD-P group. To further analyze the significantly different fecal microbial taxa between the two groups, LEfSe analysis was performed with linear discriminant analysis (LDA) threshold ≥ 3, which showed the significantly altered bacteria phylotypes. As shown in Figure 2f, the genera *Allobaculum*, *SMB53*, *Desulfovibrio* and *Clostridium* were highly enriched in the HFD-P mice, while the genera *Adlercreutzia*, *Bacteroides*, *Lactococcus*, *Streptococcus*, *Odoribacter* and *Leuconostoc* were highly enriched in the HFD-R mice. Therefore, these results indicated that the HFD-R mice reshaped the fecal microbiota composition compared to the HFD-P mice.

**Figure 3:**
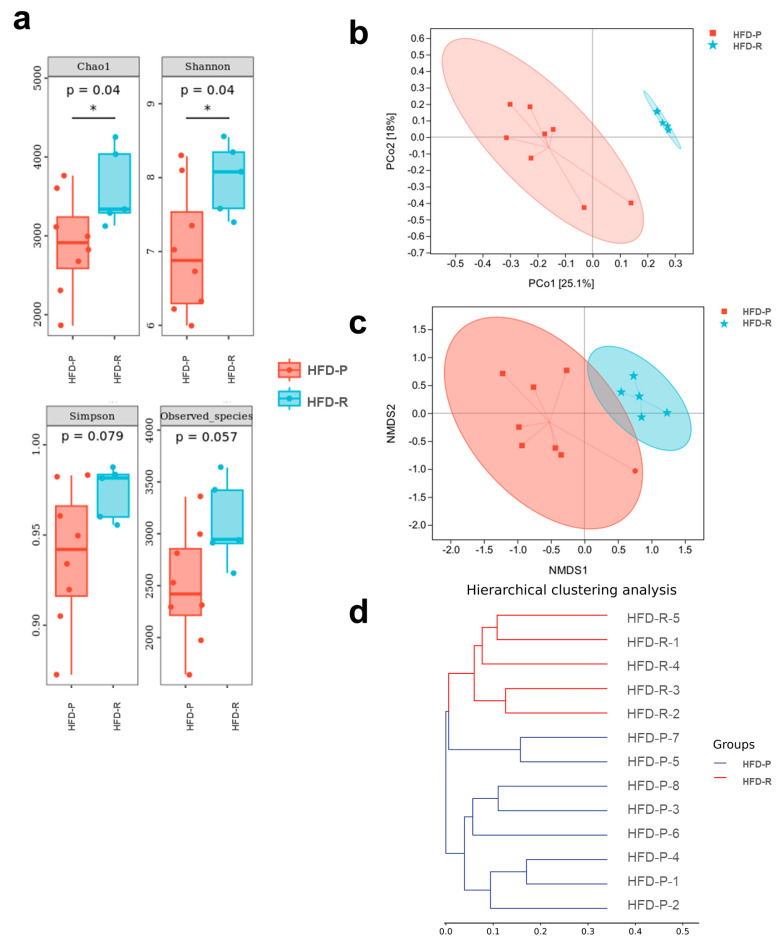
Differences in microbial diversity in feces between the obesity-prone (HFD-P) and obesity-resistant (HFD-R) mice. (**a**) Alpha diversity based on the indexes of Chao1, Shannon, Simpson and observed species. Dunn’s test was used for statistical analysis. * *p* < 0.05; (**b**) principal coordinates analysis (PCoA) plot based on Bray–Curtis distance matrices; (**c**) nonmetric multidimensional scaling (NMDS) plot based on Bray–Curtis distance matrices; (**d**) hierarchical clustering analysis.

To assess the diversity of fecal microbiota in the two groups, α-diversity was performed by the Chao 1 index, observed species, Shannon index, and Simpson index (Figure 3a). Compared to the HFD-P group, the Chao1 index and Shannon index showed a noticeable increase in the HFD-R group, whereas the Simpson index and observed species just exhibited a slight increase without statistical difference, which illustrated that the HFD-R mice increased the α-diversity to a certain extent. And the principal coordinate analysis (PCoA), nonmetric multidimensional scaling (NMDS) and hierarchical clustering analysis were carried out for the β-diversity assessment based on the Bray–Curtis distance matrices (Figure 3b–d), indicating that significant alteration of the fecal microbiota structure was made between the HFD-P group and the HFD-R group.

### 3.3. Metabolic Profiles and Variation between Obesity-Prone and Obesity-Resistant Mice by Widely Targeted Metabolomics Analysis

**Figure 4:**
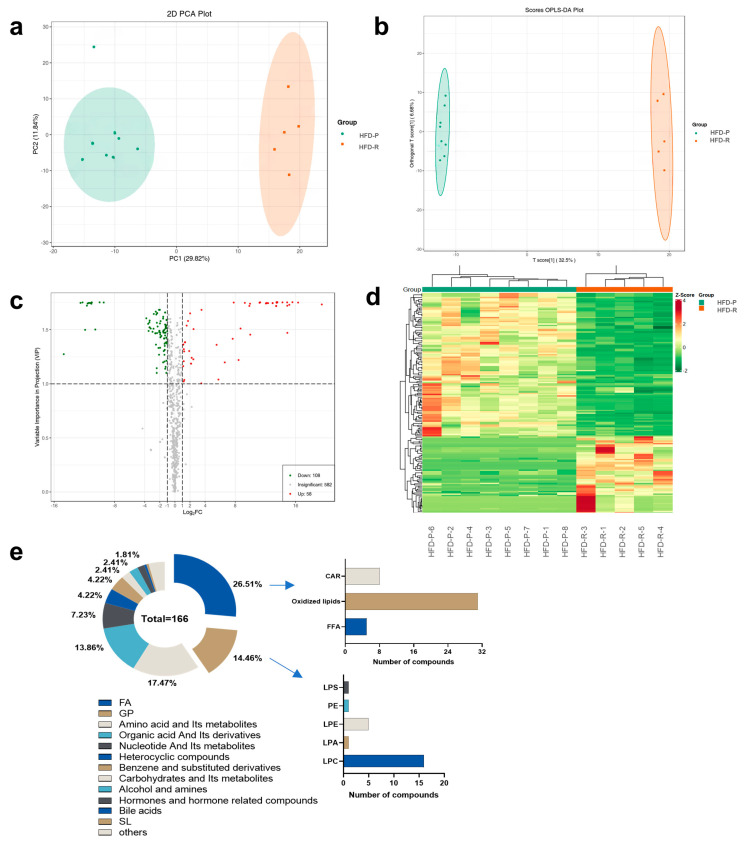
Different metabolic profiles between obesity-prone and obesity-resistant group. (**a**) PCA plot; (**b**) OPLS–DA plot; (**c**) volcano plot for differential metabolites screening; (**d**) cluster heatmap of differential metabolites; (**e**) pie chart for differential metabolites composition. fatty acyls, FA; glycerophospholipids, GP; sphingolipids, SL; CAR; free fatty acid, FFA; Lyso-phosphatidylserine, LPS; Phosphatidylethanolamine, PE; Lyso-phosphatidic acid, LPE; Lyso-phosphatidic acid, LPA; Lyso-phosphatidylcholine, LPC.

To obtain insights into the metabolic profiles and the variation in metabolites between the HFD-P group and the HFD-R group, widely targeted metabolomics analysis was used for serum metabolic profiling. Multivariate data statistical analyses were displayed to clearly exhibit the differential metabolites between the two groups. PCA plot showed that most of the samples were obviously separated into two clusters with one outlier belonging to the HFD-P group (Figure 4a). To further explore the differences of metabolic profiles, OPLS-DA analysis was carried out, revealing the dramatic metabolic differences between the two groups without any outlier (Figure 4b). To ensure the accuracy of the fitting, a permutation test (200 permutations) was performed. The R^2^ Y and Q^2^ Y values were > 0.9 and *p* < 0.005, ensuring the reliability and stability of the model (Figure S3).

VIP ≥ 1 and |Log_2_FC| ≥ 1.0 were set as the criteria for the differential metabolites screening. A total of 166 significantly altered metabolites were obtained (Figure 4c), of which 58 were up-regulated and 108 were down-regulated, compared to the metabolites in the HFD-P group. These significantly altered metabolites were able to clearly distinguish the HFD-P and the HFD-R samples by the cluster heatmap (Figure 4d). The details of these filtrated metabolites including compound name, formula, classification, CAS, VIP values, *p* values, FC and regulation types are listed in Table S1. Additionally, we counted these differential metabolites, among which fatty acyls (FA), glycerophospholipids (GP), amino acids and their metabolites, and organic acids and their derivatives were the most numerous, and the proportions were 26.51%, 14.46%, 17.47% and 13.86%, respectively (Figure 4e). Moreover, the number of secondary classified compounds in obesity-related lipids (FA and GP) were also counted and plotted in Figure 4e, where oxidized lipids were the most abundant, reaching 31, followed by lyso-phosphatidylcholine (LPC) with 16 and LPE with 5. Notably, all the significantly altered oxidized lipids containing arachidonic acid, eicosapentaenoic acid, docosahexaenoic acid, eichlerianic acid, docosatrienoic acid, docosahexaenoic acid, and their metabolites, etc., were down-regulated in the HFD-R group compared to those in the HFD-P group.

**Figure 5:**
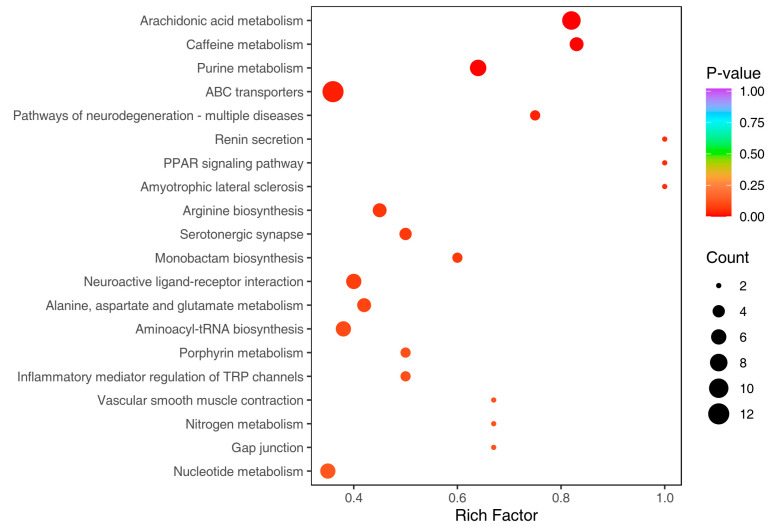
KEGG pathway enrichment analysis.

To better illustrate the relationship between the differential metabolites and the metabolic pathways, KEGG annotations were performed using compounds database, and then pathways were mapped to the KEGG database. After KEGG pathway enrichment, the top 20 significantly enriched pathways in the HFD-R mice are shown in Figure 5, i.e., AA metabolism, caffeine metabolism, purine metabolism, ABC transporters, etc. Considering the rich factor, *p* value and number of counts, the AA metabolism pathway emerged as the top 1 pathway, and was, accordingly, deemed to be the metabolic pathway with the greatest impact on the differential metabolites between the HFD-P group and the HFD-R group, providing a reference for future anti-obesity researches.

### 3.4. Obesity-Resistant Mice Altered the Metabolic Homeostasis of Arachidonic Acid

**Figure 6:**
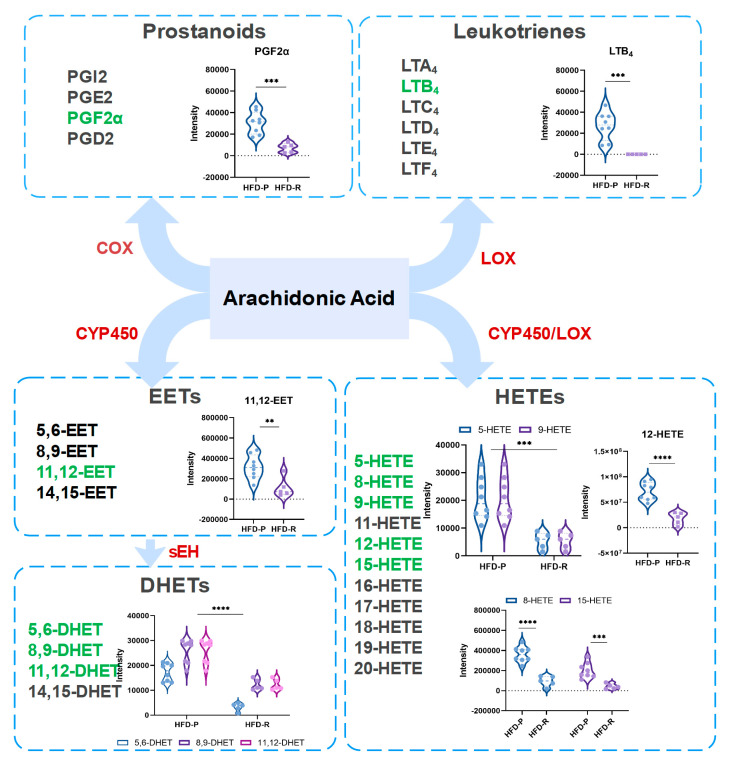
Arachidonic acid metabolism pathway analysis. Cyclooxygenase, COX; lipoxygenase, LOX; cytochrome P450, CYP450; prostanoids, PGs; leukotriene, LT; epoxyeicosatrienoic acids, EETs; dihydroxydocosatrinoic acids, DHETs; hydroxyeicosatetraenoic acids, HETEs. Unpaired *t*-test was used for statistical analysis. ** *p* < 0.01; *** *p* < 0.001; **** *p* < 0.0001.

Regarding the findings above, we further explored how the metabolic changes occurred in AA metabolism between the HFD-P group and the HFD-R group. AA is an endogenous active substance which is extensively distributed in organisms. There are three pathways for its metabolism, including cyclooxygenase (COX), lipoxygenase (LOX) and cytochrome P450 (CYP450) pathways. Figure 6 provided the whole structure of AA metabolism with the comparison of related metabolites between the two groups. For the COX pathway, AA is eventually metabolized into prostanoids (PGs), such as PGF2α, PGE2, PGI2 and PGD2, etc. In this study, only PGF2α was identified as the significantly altered metabolite related to the COX pathway. In the LOX pathway, leukotriene (LT) B4 was found to be significantly down-regulated in the HFD-R group. Furthermore, we found that metabolites of the CYP450 pathway exhibited similar changes to those of the COX and LOX pathways, i.e., 11,12–epoxyeicosatrienoic acid (11,12–EET), 5,6–dihydroxydocosatrinoic acid (5,6–DHET), 8,9–DHET, 14,15–DHET, 5-hydroxyeicosatetraenoic acids (5–HETE), 8–HETE, 9–HETE, 2–HETE and 15–HETE were down-regulated in the HFD-R mice. Taken together, eleven differential metabolites in the AA metabolism pathway were found using the widely targeted metabolomics analysis. Interestingly, all of them were decreased in the HFD-R mice. Therefore, these results provide evidence that the HFD-R mice altered the metabolic homeostasis of AA.

### 3.5. Gut Microbiota Correlated with Obesity-Related Phenotypes and Arachidonic Acid Metabolites

**Figure 7:**
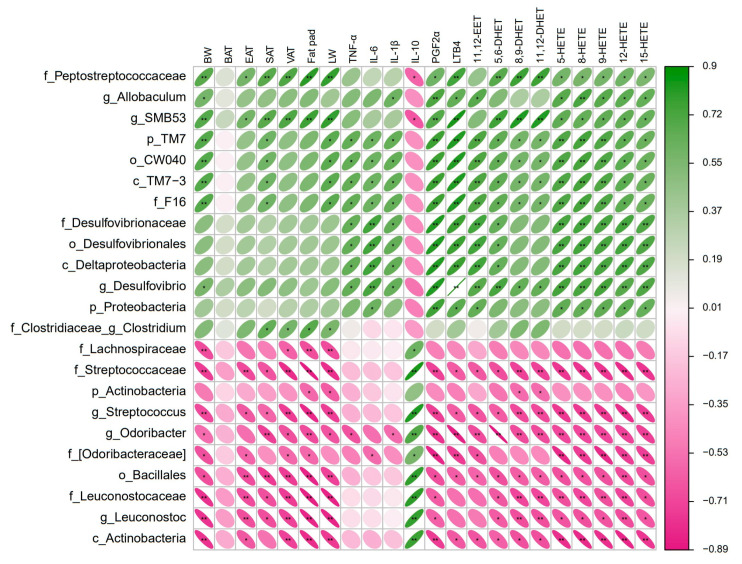
Heatmap for correlation analysis between differential microbiota and obesity-related parameters. The correlation coefficient r is shown in color. Positive correlation is shown in green; negative correlation is shown in pink. The darker the color, the stronger the correlation. * indicates *p* < 0.05, and ** indicates *p* < 0.01.

Correlations analyses between the differential microbiota and obesity-related phenotypes as well as AA metabolites were performed using Spearman’s coefficient. A total of 31 differential microbiota in both groups and 22 obesity-related indicators were correlated with coefficient r > 0.6; accordingly, 23 bacteria were found to have significant associations with most obesity-related indicators (*p* < 0.05), of which 13 were positively correlated and 10 were negatively correlated (Figure 7). Among them, there were four genera (*Allobaculumbiota*, *SMB53*, *Desulfovibrio* and *Clostridium*) positively correlated with most of the obesity-related indicators and three genera (*Streptococcus*, *Odoribacter* and *Leuconostoc*) negatively correlated with most of the obesity-related indicators. These potentially pathogenic bacteria were the dominant genera in the gut microbiota of the HFD-P mice, while in the HFD-R mice, beneficial bacteria were the dominant genera. The genera *Allobaculumbiota* was positively correlated with body weight, IL-1β and several AA metabolites. Apparently, the genera *SMB53* showed a stronger positive correlation with body weight, white adipose tissues, liver weight and AA metabolites, and a simultaneously negative correlation with the anti-inflammatory cytokine IL-10. The genera *Desulfovibrio* had the highest positive correlation with all AA metabolites and three pro-inflammatory cytokines. Instead, the genera *Streptococcus*, *Odoribacter* and *Leuconostoc* had a significantly negative association with body weight, white adipose tissues, liver weight and AA metabolites, and a positive correlation with the anti-inflammatory cytokine IL-10. These findings provide evidence that there is a strong link between microbiota changes and AA metabolism, but a cause and effect has not been demonstrated in this study, and future research is necessary.

## 4. Discussion

**Figure 8:**
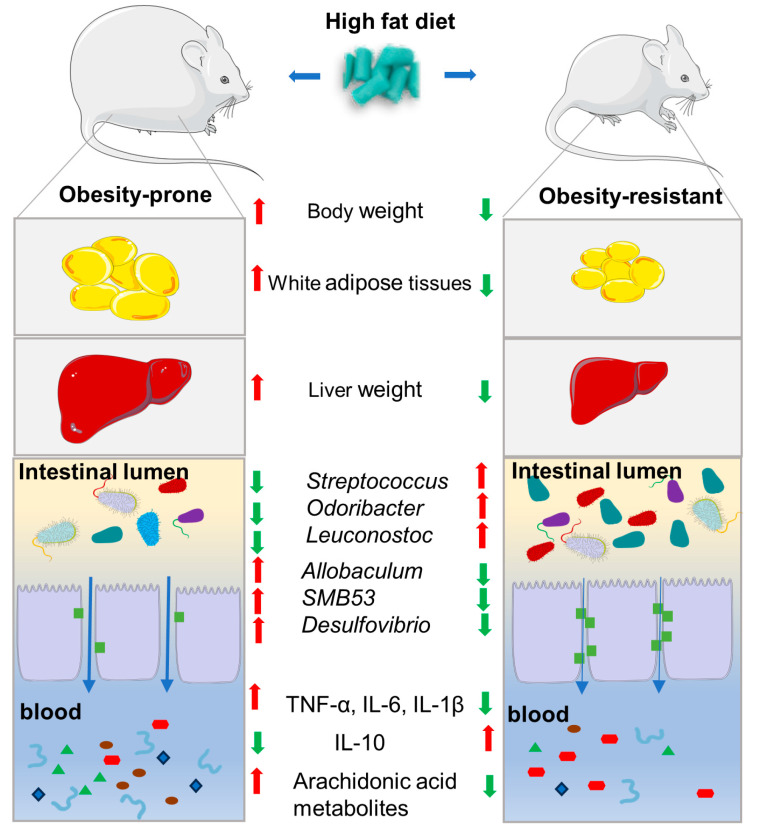
A schematical diagram of differences between obesity-prone and obesity-resistant mice. The red arrow means up-regulated; the green arrow means down-regulated.

As shown schematically in Figure 8, regarding the significant individual variations in the susceptibility to the obesity, the present study assessed the differences in physiological and biochemical indexes, fecal microbiota and serum metabolic levels between the HFD-P and the HFD-R mice with a high-fat diet.

High-fat diets are an important means of inducing obesity in mouse models. In this study, the mice with more than 1.2 times the average body weight of the control group fed chow diets were taken as the HFD-P group, while the mice with less than 1.1 times the average body weight of the control group fed chow diets were taken as the HFD-R group. Our results showed that the number of HFD-P mice was more than half of the total number of mice with high-fat diets, while the number of HFD-R mice was less than one-third. There is no uniform criteria for the evaluation of mouse susceptibility. Previous researchers believed that after induction by high-fat diets, the top one-third of mice with the highest body weight would be regarded as the HFD-P group, while the bottom one-third of mice with the lowest body weight would be regarded as the HFD-R group [19,32]. Although our data differ from these studies, they do suggest that the individual susceptibility to obesity varies, following no clear pattern.

Metabolic inflammation is considered to be an independent inflammation distinguished from traditional inflammation since it is chronic, atypical, and a low-grade state of systemic aseptic inflammation [33]. Hotamisligil et al. proposed that energy excess in nutrition was the primary factor for the development of systemic metabolic inflammation [34]. In this work, serum levels of pro-inflammatory cytokines (TNF-α, IL-6 and IL-1β) in the HFD-R mice were significantly lower than those in the HFD-P mice, while the diametrical results of anti-inflammatory cytokine IL-10 were observed. This may be due to the fact that in response to obesity, adipocytes secrete these pro-inflammatory cytokines to reduce lipids content [35]. With an increasing degree of obesity, the level of free fatty acids in the blood also increases correspondingly, and they are deposited in the liver and other organs ectopically through the blood circulation, thus producing lipotoxicity and further promoting systemic inflammation [36].

Previous studies have shown that the structural composition of the gut microbiota is related to the host phenotype of obesity or lean [16,20,37]. Although the causal relationship between the abundance of specific bacteria and obesity is not fully understood, changes in the gut microbiota composition have been confirmed. The results of microbiome analysis in this study showed that the composition of fecal microbiota and the biodiversity of the HFD-R mice were significantly increased compared to the HFD-P mice, which is in-line with most of the reported data [38,39,40] and contrary to Kasai et al. [41], who found that humans with obesity have greater biodiversity. The present study found that *Firmicutes* and *Bacteroidetes* were the most abundant bacterial phyla in both groups of mice, but the levels of *Firmicutes*, *Bacteroidetes* and their ratios *F/B* were not significantly different between the two groups, which is in-line with the results in previously reported studies [18,42]. Consequently, the accuracy of a lower *F/B* ratio as an indicator of the HFD-R group still remains controversial, suggesting that the phenotypes of HFD-P and HFD-R cannot be distinguished by simple comparison of the the relative abundance at the phylum level. Accordingly, differences at more specific taxa, like genus or even species, levels seem to be related to changes in metabolic function [43]. This study found that the genera *Streptococcus*, *Odoribacter*, and *Leuconostoc* were significantly enriched in the HFD-R mice, while the genera *Allobaculum*, *SMB53*, *Desulfovibrio* and *Clostridium* were significantly decreased. Among them, the significant increases in *Odoribacter* and decreases in *Allobaculum* and *SMB53* were consistent with the publication of Li et al. [44]. *Odoribacter*, a common probiotic, can produce short-chain fatty acids, reduce inflammation [45], and also eliminate succinic acid and improve the body’s glucose tolerance [46]. *Streptococcus* in cecum contents of obese piglets induced by high-energy diets was also significantly diminished [47], while in contrast, it was significantly enriched in obese mice induced by high-fat diets, as reported by Yu et al. [48]. Currently, there are few studies on the roles of *Streptococcus* in anti-obesity, and the mechanism of action is still unclear; therefore, further confirmation is required. Petersen et al. [49] found that the proliferation of *Desulfovibrio* and the loss of *Clostridia* were the key features of obesity, and *Desulfovibrio* was capable of producing lipopolysaccharide in the blood to activate TLR4-dependent (Toll-like receptor 4) signaling and aggravate inflammation and insulin resistance.

Obesity is often accompanied by disorders of host metabolism [50], such as lipids peroxidation, phospholipids metabolism, glucose metabolism, and amino acids metabolism etc.; however, the effects of lipids on obesity are still unclear. Considering the broad biological role of lipids such as signaling molecules, biofilm composition and energy storage, lipids studies are crucially important. In this study, widely targeted metabolomics analysis showed that oxidized lipids were the most differential metabolites between the HFD-P group and the HFD-R group, especially for AA metabolites. AA, as the derivative of linoleic acid, is one of the crucial n-6 fatty acids. However, few studies investigated the relationship among AA metabolism, obesity, and the gut microbiota. It is well known that AA metabolites are highly active inflammatory mediators, which is in-line with our results that the HFD-R mice down-regulated 11 AA metabolites and showed lower levels of pro-inflammatory cytokines. In addition, a previous report found that AA affected obesity via the gut–hypothalamus–adipose–liver axis, i.e., AA could aggravate obesity, enhance the proliferation of pro-inflammatory microbiota, exacerbate nonalcoholic steatohepatitis and induce insulin resistance [51]. Interestingly, this study found that all significantly altered AA metabolites were positively correlated with the dominant genera in the HFD-P group and negatively correlated with those in the HFD-R group. Furthermore, AA metabolites were also significantly increased in obese pigs rather than in lean pigs [52]. Based on the above results, we can tentatively consider that a reduction in AA metabolites may be a marker of obesity-resistant individuals.

In this study, we obtained differential bacteria and differential AA metabolites that could distinguish the HFD-P and the HFD-R mice, which would provide new insights into further understanding individual variations in the susceptibility to obesity. However, limitations of this study should be noted to facilitate further research. Firstly, parameters changed at week 16, such as gut microbiota, cytokines and AA metabolites, were not evaluated to establish whether the changes were not present at week 1. Secondly, differences in the above parameters were found between the HFD-P and the HFD-R mice, and whether these parameters could really affect the susceptibility of mice to obesity was not verified. Therefore, further studies such as the colonization of specific bacteria and intervention with specific differential metabolites still need to be conducted. Thirdly, the differences in intestinal nutrients absorption between the HFD-P and the HFD-R mice were not evaluated, which might explain their differences in body weight. Bomb calorimetry, a gold standard for the assessment of intestinal absorption capacity, could indicate whether there is a nutrient absorption defect in HFD-R mice. Whether possible intestinal absorption defects are related to changes in the gut microbiota and AA metabolites is also unknown. All these aspects mentioned above provide ideas for our future research. It is worth noting that the findings in this work provide a reference for the prevention and treatment of diet-induced obesity in mice. It is indicated that the HFD-P and the HFD-R subtypes may occur during the study of HFD-induced obesity using C57BL/6J, suggesting that researchers need to pay particular attention to the potential effects of this situation on their research results.

## 5. Conclusions

Taken together, this study demonstrated the HFD-R mice showed lower body weight, liver weight, adipose accumulation and pro-inflammatory cytokines compared to the HFD-P mice. Also, the composition and biodiversity of the fecal microbiota were different between the two groups. The genera *Allobaculumbiota*, *SMB53*, *Desulfovibrio* and *Clostridium* increased in the HFD-P mice, and the genera *Streptococcus*, *Odoribacter* and *Leuconostoc* were enriched in the HFD-R mice. A total of 166 differential metabolites were found by widely targeted metabolomics analysis, especially those products involved in AA metabolism, which were significantly decreased in the HFD-R mice. Moreover, KEGG pathway analysis exhibited that AA metabolism was the most enriched pathway. Significantly altered bacteria and obesity-related parameters as well as AA metabolites exhibited strong correlation. Overall, the phenotypes of HFD-P and HFD-R were linked to the gut microbiota and AA metabolism.

## Data Availability

Data are contained within the article and Supplementary Materials.

## Supplementary Materials

The following supporting information can be downloaded at: https://www.mdpi.com/article/10.3390/nu16111579/s1, Figure S1: Ratios of different tissue weights to body weights; Figure S2: Relative abundance of *Firmicutes*, *Bacteroidetes* and *Firmicutes/Bacteroidetes* (*F/B*) ratio; Figure S3: The permutation test for OPLS-DA analysis; Table S1: The differential metabolites between the obesity-prone and the obesity-resistant mice (HFD-R vs. HFD-P).
