# Supplementary material for: Distinct Gut Microbiota and Arachidonic Acid Metabolism in Obesity-Prone and Obesity-Resistant Mice with a High-Fat Diet
Source: Nutrients. 2024 May 23;16(11):1579. doi: 10.3390/nu16111579 (PMC11174461; doi:10.3390/nu16111579)
Supplement: Supplementary file 1 [file nutrients-16-01579-s001.zip › nutrients-2974239-supplementary.pdf]

Distinct Gut Microbiota and Arachidonic Acid Metabolism in Obesity-prone and Obesity-resistant Mice with a High-Fat Diet

Huixia Zhang, Shiqi Chen, Liu Yang, Shuai Zhang, Linqian Qin and Haiyang Jiang*

National Key Laboratory of Veterinary Public Health and Safety, Department of Veterinary Pharmacology and Toxicology, College of Veterinary Medicine, China Agricultural University, Beijing 100193, China

* Correspondence: haiyang@cau.edu.cn; Tel: +86-010-62734478; Fax: +86-010-62731032

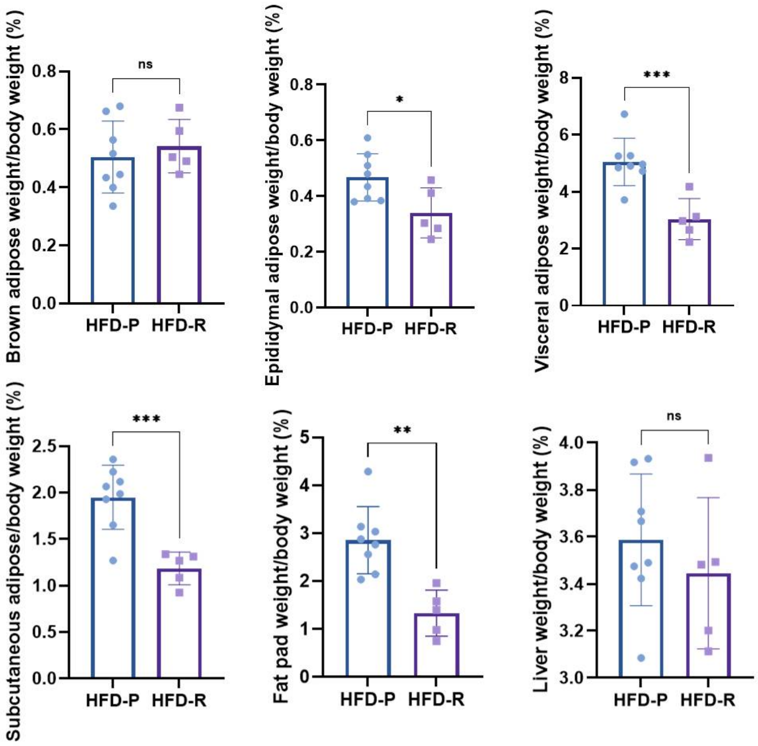

Figure S1. Ratios of different tissue weights tobody weights. Data was presented as means±SD. Statistical analysis was used unpaired t-test. ***P<0.001.

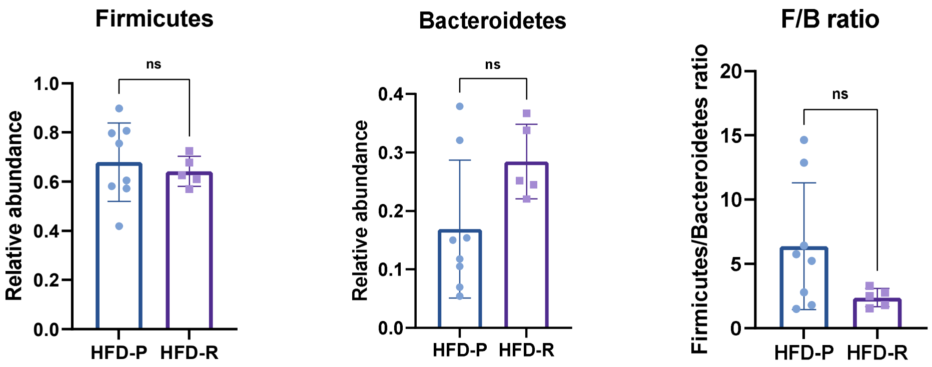

Figure S2. Relative abundance of *Firmicutes*, *Bacteroidetes* and *Firmicutes*/*Bacteroidetes* (*F*/*B*) ratio. Data was presented as means±SD. Statistical analysis was used unpaired *t*-test. *ns* indicates no significant difference.

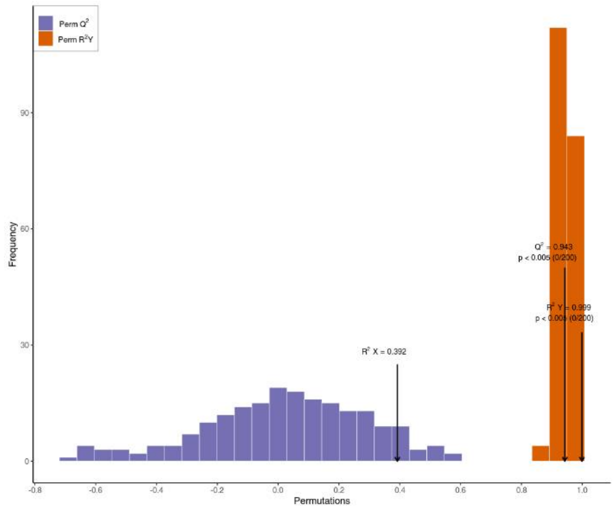

Figure S3. The permutation test for OPLS-DA analysis.

Table S1. The differential metabolites between the obesity-prone and the obesity-resistant mice (HFD-R vs HFD-P).

Compounds	Formula	Class I	Class II	CAS	VIP	P-value	Fold_Change	Log2FC	Type
(3-Methoxy-4-hydroxyphenyl)ethylene glycol sulfate	C9H12O7S	Organic acid And Its derivatives	Organic acid And Its derivatives	3415-67-6	1.754	0.00040	11691.830	13.513	up
(E)-3-(3-hydroxy-4-methoxyphenyl)acrylic acid	C10H10O4	Benzene and substituted derivatives	Benzene and substituted derivatives	25522-33-2	1.749	0.00345	2390.021	11.223	up
(R)-(-)-2-Phenylpropionic Acid	C9H10O2	Organic acid And Its derivatives	Organic acid And Its derivatives	7782-26-5	1.743	0.02439	4285.706	12.065	up
(±)11(12)-DiHET	C20H34O4	FA	Oxidized lipids	-	1.588	0.00003	0.476	-1.071	down
(±)12-HEPE	C20H30O3	FA	Oxidized lipids	81187-21-5	1.521	0.00059	0.098	-3.353	down
(±)12-HETE	C20H32O3	FA	Oxidized lipids	71030-37-0	1.333	0.00006	0.258	-1.954	down
(±)15-HEPE	C20H30O3	FA	Oxidized lipids	88852-33-9	1.521	0.00059	0.098	-3.353	down
(±)15-HETE	C20H32O3	FA	Oxidized lipids	54845-95-3	1.529	0.00038	0.205	-2.288	down
(±)17-HDHA	C22H32O3	FA	Oxidized lipids	90780-52-2	1.330	0.00338	0.222	-2.173	down
(±)18-HEPE	C20H30O3	FA	Oxidized lipids	141110-17-0	1.521	0.00059	0.098	-3.353	down
(±)5-HETE	C20H32O3	FA	Oxidized lipids	70608-72-9	1.430	0.00057	0.265	-1.916	down
(±)8(9)-DiHET	C20H34O4	FA	Oxidized lipids	192461-96-4	1.588	0.00003	0.476	-1.071	down
(±)8-HETE	C20H32O3	FA	Oxidized lipids	98462-03-4	1.455	0.00001	0.260	-1.945	down
(±)9-HETE	C20H32O3	FA	Oxidized lipids	79495-85-5	1.430	0.00057	0.265	-1.916	down
1-Aminocyclobutanecarboxylic acid	C5H9NO2	Amino acid and Its metabolites	Amino acid derivatives	22264-50-2	1.514	0.00050	0.447	-1.163	down
1-Methyluric Acid	C6H6N4O3	Amino acid and Its metabolites	Amino acid derivatives	708-79-2	1.746	0.03752	7918.087	12.951	up

10-HDoHE	C22H32O3	FA	Oxidized lipids	90780-50-0	1.480	0.00070	0.128	-2.963	down
11,12-EET	C20H32O3	FA	Oxidized lipids	123931-40-8	1.302	0.00723	0.358	-1.480	down
11-HDoHE	C22H32O3	FA	Oxidized lipids	87018-59-5	1.585	0.00026	0.099	-3.342	down
11β-13,14-dihydro-15-keto Prostaglandin F2α	C20H34O5	FA	Oxidized lipids	107615-77-0	1.489	0.00021	0.240	-2.058	down
12-OxoETE	C20H30O3	FA	Oxidized lipids	108437-64-5	1.101	0.06998	0.189	-2.406	down
13(R)-HODE	C18H32O3	FA	Oxidized lipids	10219-69-9	1.509	0.00585	0.141	-2.831	down
13,14-Dihydro-15-keto Prostaglandin F2α	C20H34O5	FA	Oxidized lipids	27376-76-7	1.489	0.00021	0.240	-2.058	down
13-HDoHE	C22H32O3	FA	Oxidized lipids	90780-53-3	1.751	0.00009	0.000	-11.039	down
13-HOTrE	C18H30O3	FA	Oxidized lipids	87984-82-5	1.740	0.00234	0.000	-11.070	down
14(S)-HDHA	C22H32O3	FA	Oxidized lipids	119433-37-3	1.554	0.00078	0.086	-3.541	down
15(S)-HETrE	C20H34O3	FA	Oxidized lipids	92693-02-2	1.466	0.00079	0.166	-2.587	down
15-oxoETE	C20H30O3	FA	Oxidized lipids	81416-72-0	1.751	0.00003	0.001	-9.434	down
16-HDoHE	C22H32O3	FA	Oxidized lipids	90780-51-1	1.488	0.00108	0.090	-3.481	down
18-Hydroxycorticosterone	C21H30O5	Hormones and hormone related compounds	Hormones and hormone related compounds	561-65-9	1.722	0.01006	0.000	-11.665	down
2,2-Dimethylglutaric acid	C7H12O4	Organic acid And Its derivatives	Organic acid And Its derivatives	681-57-2	1.362	0.04080	2.159	1.110	up
2,6-Dimethoxybenzoic acid	C9H10O4	Organic acid And Its derivatives	Organic acid And Its derivatives	1466-76-8	1.746	0.00016	0.000	-11.239	down
2-(Dimethylamino)Guanosine	C12H17N5O5	Nucleotide And Its metabolites	Nucleotide And Its metabolites	2140-67-2	1.498	0.00474	0.380	-1.396	down

2-(Formylamino)Benzoic Acid	C8H7NO3	Benzene and substituted derivatives	Phenolic acids	3342-77-6	1.537	0.01274	2.945	1.558	up
2-Methyl-d-erythritol 2,4-cyclodiphosphate	C5H12O9P2	Carbohydrates and Its metabolites	Phosphate sugars	143488-44-2	1.470	0.03919	30689.926	14.905	up
2-Phenylbutyric acid	C10H12O2	Organic acid And Its derivatives	Organic acid And Its derivatives	90-27-7	1.753	0.00065	11349.712	13.470	up
2-Phenylpropylamine	C9H13N	Benzene and substituted derivatives	Benzene and substituted derivatives	582-22-9	1.748	0.00014	0.000	-11.398	down
2-pyrimidine acetic acid	C6H6N2O2	Heterocyclic compounds	Heterocyclic compounds	66621-73-6	1.754	0.00129	48982.340	15.580	up
3-Amino-4-Hydroxybenzoic Acid	C7H7NO3	Benzene and substituted derivatives	Phenolic acids	1571-72-8	1.719	0.18538	10991.539	13.424	up
3-Amino-5-hydroxybenzoic acid	C7H7NO3	Organic acid And Its derivatives	Organic acid And Its derivatives	76045-71-1	1.719	0.18538	10991.539	13.424	up
3-Carboxypropyltrimethylammonium	C7H15NO2	Organic acid And Its derivatives	Organic acid And Its derivatives	6249-56-5	1.302	0.01011	3.722	1.896	up
3-Hydroxy-3-Methylpentane-1,5-Dioic Acid	C6H10O5	Amino acid and Its metabolites	Amino acid derivatives	503-49-1	1.162	0.09597	2.148	1.103	up
3-Hydroxyanthranilic Acid	C7H7NO3	Benzene and substituted derivatives	Phenolic acids	548-93-6	1.719	0.18538	10991.539	13.424	up
3-Hydroxycinnamic acid	C9H8O3	Organic acid And Its derivatives	Organic acid And Its derivatives	14755-02-3	1.683	0.00064	11.546	3.529	up

3-Methyladenine	C6H7N5	Nucleotide And Its metabolites	Nucleotide And Its metabolites	5142-23-4	1.747	0.00087	0.000	-12.509	down
3-Methyladipic acid	C7H12O4	Organic acid And Its derivatives	Organic acid And Its derivatives	3058-01-3	1.362	0.04080	2.159	1.110	up
3-Sulfocatechol	C6H6O5S	Benzene and substituted derivatives	Phenolics	35857-70-6	1.225	0.16388	5.450	2.446	up
3-aminobenzamide	C7H8N2O	Benzene and substituted derivatives	Benzene and substituted derivatives	3544-24-9	1.721	0.19141	30488.971	14.896	up
4-Acetylaminobenzoic acid	C9H9NO3	Organic acid And Its derivatives	Organic acid And Its derivatives	556-08-1	1.186	0.16138	4.412	2.141	up
4-acetoxyphenol	C8H8O3	Benzene and substituted derivatives	Phenolics	3233-32-7	1.510	0.23247	417.287	8.705	up
4-toluenesulfonic acid	C7H7SO3 H	Organic acid And Its derivatives	Organic acid And Its derivatives	104-15-4	1.753	0.00052	2530.979	11.305	up
5'-Deoxy-5'-(Methylthio) Adenosine	C11H15N5 O3S	Nucleotide And Its metabolites	Nucleotide And Its metabolites	2457-80-9	1.298	0.00772	2.331	1.221	up
5,6-DiHETrE	C20H34O4	FA	Oxidized lipids	213382-49-1	1.563	0.00000	0.191	-2.388	down
5-HETrE	C20H34O3	FA	Oxidized lipids	195061-94-0	1.466	0.00079	0.166	-2.587	down
5-Methoxytryptamine	C11H14N2 O	Alcohol and amines	Amines	608-07-1	1.023	0.10697	2.252	1.171	up
5-hydroxyindole-2-carboxylic acid	C9H7NO3	Heterocyclic compounds	Indole and Its derivatives	21598-06-1	1.683	0.00006	0.231	-2.112	down
5-nitrobenzimidazole	C7H5N3O 2	Heterocyclic compounds	Heterocyclic compounds	94-52-0	1.752	0.00004	0.000	-12.238	down

6-Methylnicotinamide	C7H8N2O	Heterocyclic compounds	Pteridines and derivatives	6960-22-1	1.005	0.25166	11.381	3.509	up
6-trans-12-epi Leukotriene B4	C20H32O4	FA	Oxidized lipids	71548-19-1	1.742	0.00070	0.000	-11.557	down
7-Methyluric Acid	C6H6N4O3	Amino acid and Its metabolites	Amino acid derivatives	612-37-3	1.746	0.03752	7918.087	12.951	up
8(S)-HETrE	C20H34O3	FA	Oxidized lipids	889573-69-7	1.466	0.00079	0.166	-2.587	down
8,8a-deoxy-oleane	C20H36O6	Others	Others	53428-54-9	1.752	0.00233	19606.428	14.259	up
8-HDoHE	C22H32O3	FA	Oxidized lipids	90780-54-4	1.510	0.00071	0.108	-3.205	down
Acetylcholine	C7H15NO2	Alcohol and amines	Polyamines	60-31-1	1.580	0.00213	3.341	1.740	up
Acrylamide	C3H5NO	Alcohol and amines	Amines	79-06-1	1.498	0.00182	0.000	-11.895	down
Adenosine	C10H13N5O4	Nucleotide And Its metabolites	Nucleotide And Its metabolites	58-61-7	1.650	0.00666	4.044	2.016	up
Allopurinol	C5H4N4O	Nucleotide And Its metabolites	Nucleotide And Its metabolites	315-30-0	1.730	0.20418	36744.979	15.165	up
Androsterone sulfate	C19H30O5S	Hormones and hormone related compounds	Hormones and hormone related compounds	2479-86-9	1.751	0.00067	222.167	7.796	up
Arg-Val	C11H23N5O3	Amino acid and Its metabolites	Small Peptide	2896-20-0	1.380	0.00629	0.420	-1.251	down
Butenoyl-PAF	C28H56NO7P	Others	Others	-	1.472	0.00094	0.421	-1.250	down
Caffeine	C8H10N4O2	Heterocyclic compounds	Heterocyclic compounds	58-08-2	1.719	0.07359	935.205	9.869	up

Carbamoyl phosphate	CH4NO5P	Organic acid And Its derivatives	Phosphoric acids	590-55-6	1.313	0.00153	0.392	-1.352	down
Carnitine C10:1	C17H31N O4	FA	CAR	-	1.748	0.01258	618.000	9.271	up
Carnitine C11:DC	C18H33N O6	FA	CAR	-	1.472	0.00214	0.259	-1.947	down
Carnitine C12-OH	C19H37N O5	FA	CAR	-	1.477	0.00256	0.237	-2.074	down
Carnitine C14-OH	C21H41N O5	FA	CAR	-	1.361	0.01024	0.175	-2.511	down
Carnitine C14:1	C21H39N O4	FA	CAR	-	1.267	0.02429	0.384	-1.382	down
Carnitine C16-OH	C23H45N O5	FA	CAR	195207-76-2	1.579	0.00251	0.269	-1.897	down
Carnitine C18:1-OH	C25H47N O5	FA	CAR	-	1.349	0.01660	0.393	-1.346	down
Carnitine C8-OH	C15H29N O5	FA	CAR	-	1.441	0.00617	0.289	-1.792	down
Choline	C5H14NO +	Tryptamines,Cholines, Pigments	Cholines	62-49-7	1.425	0.00083	0.456	-1.132	down
Cyclo(Phe-Pro)	C14H16N2 O2	Amino acid and Its metabolites	Small Peptide	14705-60-3	1.539	0.00016	0.485	-1.043	down
Cyclo(Pro-Phe)	C14H16N2 O2	Amino acid and Its metabolites	Small Peptide	3705-26-8	1.539	0.00016	0.485	-1.043	down
D-Calcium Pantothenate	C18H34N2 O10	CoEnzyme and vitamins	CoEnzyme and vitamins	137-08-6	1.505	0.03123	5.544	2.471	up

D-Mannitol	C6H14O6	Carbohydrates and Its metabolites	Sugar alcohols	69-65-8	1.475	0.00048	0.348	-1.522	down
D-Sorbitol	C6H14O6	Carbohydrates and Its metabolites	Sugar alcohols	50-70-4	1.475	0.00048	0.348	-1.522	down
DHA	C22H32O2	FA	Oxidized lipids	6217-54-5	1.545	0.00022	0.420	-1.251	down
DL-2-hydroxystearic acid	C18H36O3	FA	FFA	629-22-1	1.446	0.00161	0.407	-1.296	down
Dl-Glyceraldehyde 3-Phosphate	C3H7O6P	Organic acid And Its derivatives	Phosphoric acids	591-59-3	1.750	0.03379	82646.048	16.335	up
Dulcitol	C6H14O6	Carbohydrates and Its metabolites	Sugar alcohols	608-66-2	1.683	0.00001	0.251	-1.995	down
Ectoine	C6H10N2 O2	Heterocyclic compounds	Heterocyclic compounds	96702-03-3	1.219	0.10713	347.241	8.440	up
FAHFA(8:0/10:0)	C18H34O4	FA	FFA	-	1.752	0.00002	0.000	-11.012	down
FFA(22:6)	C22H32O2	FA	FFA	25167-62-8	1.511	0.00026	0.422	-1.246	down
FFA(22:7)	C22H30O2	FA	FFA	-	1.567	0.00052	0.096	-3.379	down
Ferulic acid	C10H10O4	Organic acid And Its derivatives	Organic acid And Its derivatives	537-98-4	1.749	0.00345	2390.021	11.223	up
Gly-Val	C7H14N2 O3	Amino acid and Its metabolites	Small Peptide	1963-21-9	1.605	0.00813	0.073	-3.786	down
Guanosine	C10H13N5 O5	Nucleotide And Its metabolites	Nucleotide And Its metabolites	118-00-3	1.745	0.09157	5252.505	12.359	up
Hippuric Acid	C9H9NO3	Organic acid And Its derivatives	Organic acid And Its derivatives	495-69-2	1.186	0.16138	4.412	2.141	up
Hydrocinnamic Acid	C9H10O2	Organic acid And Its derivatives	Organic acid And Its derivatives	501-52-0	1.743	0.02439	4285.706	12.065	up

Hydroquinone	C6H6O2	Benzene and substituted derivatives	Phenolics	123-31-9	1.240	0.15838	4.677	2.225	up
Hypoxanthine	C5H4N4O	Nucleotide And Its metabolites	Nucleotide And Its metabolites	68-94-0	1.415	0.31127	202.094	7.659	up
Hypoxanthine-9-β-D-Arabinofuranoside	C10H12N4O5	Nucleotide And Its metabolites	Nucleotide And Its metabolites	7013-16-3	1.040	0.19891	54.645	5.772	up
Ile-Pro-Ile	C17H31N3O4	Amino acid and Its metabolites	Small Peptide	90614-48-5	1.372	0.00306	0.477	-1.069	down
Iminodiacetic acid	C4H7NO4	Organic acid And Its derivatives	Organic acid And Its derivatives	142-73-4	1.640	0.00035	0.182	-2.460	down
Indoxylsulfuric acid	C8H7NO4S	Heterocyclic compounds	Indole and Its derivatives	487-94-5	1.319	0.01844	2.273	1.184	up
Inosine	C10H12N4O5	Nucleotide And Its metabolites	Nucleotide And Its metabolites	58-63-9	1.360	0.21710	46.160	5.529	up
Isocitric acid	C6H8O7	Organic acid And Its derivatives	Organic acid And Its derivatives	320-77-4	1.205	0.01226	0.387	-1.370	down
L-Arginine	C6H14N4O2	Amino acid and Its metabolites	Amino acids	74-79-3	1.138	0.01869	0.411	-1.283	down
L-Aspartic Acid	C4H7NO4	Amino acid and Its metabolites	Amino acids	56-84-8	1.645	0.00072	0.144	-2.795	down
L-Cysteine	C3H7NO2S	Amino acid and Its metabolites	Amino acids	52-90-4	1.212	0.00802	0.460	-1.121	down
L-Glutamic Acid	C5H9NO4	Amino acid and Its metabolites	Amino acids	56-86-0	1.653	0.00005	0.197	-2.345	down
L-Proline	C5H9NO2	Amino acid and Its metabolites	Amino acids	147-85-3	1.514	0.00050	0.447	-1.163	down

L-Threonine	C4H9NO3	Amino acid and Its metabolites	Amino acids	72-19-5	1.268	0.03729	0.233	-2.103	down
L-threo-3-Methylaspartate	C5H9NO4	Amino acid and Its metabolites	Amino acid derivatives	6061-13-8	1.653	0.00005	0.197	-2.345	down
LPA(0:0/18:0)	C21H43O7P	GP	LPC	-	1.369	0.00085	0.388	-1.364	down
LPA(16:0/0:0)	C19H39O7P	GP	LPA	22002-85-3	1.385	0.00159	0.477	-1.069	down
LPC(0:0/14:0)	C22H46NO7P	GP	LPC	20559-16-4	1.525	0.00025	0.431	-1.215	down
LPC(0:0/16:1)	C24H48NO7P	GP	LPC	-	1.528	0.00020	0.433	-1.207	down
LPC(0:0/18:0)	C26H54NO7P	GP	LPC	-	1.426	0.00042	0.457	-1.130	down
LPC(0:0/20:1)	C28H56NO7P	GP	LPC	-	1.472	0.00094	0.421	-1.250	down
LPC(0:0/20:2)	C28H54NO7P	GP	LPC	-	1.590	0.00011	0.464	-1.107	down
LPC(0:0/20:3)	C28H52NO7P	GP	LPC	-	1.546	0.00010	0.409	-1.290	down
LPC(12:0/0:0)	C20H42NO7P	GP	LPC	-	1.501	0.00073	0.001	-10.547	down
LPC(16:1/0:0)	C24H48NO7P	GP	LPC	-	1.528	0.00020	0.433	-1.207	down
LPC(16:2/0:0)	C24H46NO7P	GP	LPC	-	1.491	0.00164	0.350	-1.514	down

LPC(18:0/0:0)	C26H54NO7P	GP	LPC	-	1.426	0.00042	0.457	-1.130	down
LPC(20:1/0:0)	C28H56NO7P	GP	LPC	-	1.472	0.00094	0.421	-1.250	down
LPC(20:2/0:0)	C28H54NO7P	GP	LPC	-	1.590	0.00011	0.464	-1.107	down
LPC(20:3/0:0)	C28H52NO7P	GP	LPC	-	1.546	0.00010	0.409	-1.290	down
LPC(O-16:0/2:0)	C26H54NO7P	GP	LPC	74389-68-7	1.426	0.00042	0.457	-1.130	down
LPC(O-18:1/0:0)	C26H54NO6P	GP	LPC	87907-66-2	1.471	0.00057	0.386	-1.375	down
LPE(0:0/20:2)	C25H48NO7P	GP	LPE	-	1.658	0.00005	0.407	-1.296	down
LPE(14:0/0:0)	C19H40NO7P	GP	LPE	-	1.473	0.00040	0.355	-1.494	down
LPE(17:1/0:0)	C22H44NO7P	GP	LPE	-	1.752	0.00001	0.001	-9.978	down
LPE(20:2/0:0)	C25H48NO7P	GP	LPE	-	1.658	0.00005	0.407	-1.296	down
LPS(22:6/0:0)	C28H44NO9P	GP	LPS	-	1.197	0.08180	97.686	6.610	up
LTB4	C20H32O4	FA	Oxidized lipids	71160-24-2	1.742	0.00070	0.000	-11.557	down
Lythramine	C29H37NO5	Alcohol and amines	Polyamines	32420-56-7	1.418	0.00248	0.465	-1.104	down

M-toluene acetic acid	C9H10O2	Organic acid And Its derivatives	Organic acid And Its derivatives	621-36-3	1.747	0.01458	4657.510	12.185	up
N-(1-Deoxy-1-fructosyl)phenylalanine	C15H21NO7	Amino acid and Its metabolites	Amino acid derivatives	87251-83-0	1.602	0.00006	0.339	-1.560	down
N-Acetyl-Asp-Glu	C11H16N2O8	Amino acid and Its metabolites	Amino acid derivatives	3106-85-2	1.208	0.10373	2.259	1.176	up
N-Acetylaspartate	C6H9NO5	Amino acid and Its metabolites	Amino acid derivatives	997-55-7	1.382	0.02341	2.531	1.339	up
N-Acetylglycine	C4H7NO3	Amino acid and Its metabolites	Amino acid derivatives	543-24-8	1.384	0.00621	2.548	1.349	up
N-Amidino-L-Aspartate	C5H9N3O4	Amino acid and Its metabolites	Amino acid derivatives	6133-30-8	1.156	0.02039	0.426	-1.231	down
N-Cinnamylglycine	C11H11NO3	Organic acid And Its derivatives	Organic acid And Its derivatives	16534-24-0	1.748	0.01306	11624.993	13.505	up
N-acetylornithine	C7H14N2O3	Amino acid and Its metabolites	Amino acid derivatives	6205-08-9	1.197	0.01203	0.339	-1.562	down
N-lactoyl-phenylalanine	C12H15NO4	Amino acid and Its metabolites	Amino acid derivatives	-	1.667	0.00010	0.173	-2.534	down
N6-(2-Hydroxyethyl)adenosine	C12H17N5O5	Nucleotide And Its metabolites	Nucleotide And Its metabolites	4338-48-1	1.484	0.00202	0.341	-1.554	down
Norepinephrine	C8H11NO3	Hormones and hormone related compounds	Hormones and hormone related compounds	51-41-2	1.101	0.01175	0.456	-1.134	down
O-Phospho-L-threonine	C4H10NO6P	Amino acid and Its metabolites	Amino acid derivatives	1114-81-4	1.420	0.00287	0.375	-1.416	down

O-Phosphorylethanolamine	C2H8NO4P	GP	PE	1071-23-4	1.313	0.00153	0.392	-1.352	down
Phosphatidylethanolamine lyso alkenyl 18:2	C23H44NO6P	GP	LPE	-	1.619	0.00001	0.342	-1.548	down
Pimelic acid	C7H12O4	Organic acid And Its derivatives	Organic acid And Its derivatives	111-16-0	1.362	0.04080	2.159	1.110	up
Pinolenic acid	C18H30O2	FA	FFA	16833-54-8	1.485	0.00023	0.496	-1.011	down
Pyrophosphate	H4O7P2	Organic acid And Its derivatives	Phosphoric acids	2466-09-3	1.468	0.00070	0.195	-2.357	down
ST-638	C19H18N2O3S	Organic acid And Its derivatives	Organic acid And Its derivatives	107761-24-0	1.740	0.01983	994.689	9.958	up
Salicylaldehyde	C7H6O2	Benzene and substituted derivatives	Benzene and substituted derivatives	90-02-8	1.752	0.00596	30125.235	14.879	up
Sphingosyl-phosphocholine	C23H50N2O5P	SL	SM	-	1.417	0.00088	0.370	-1.436	down
Taurolithocholic acid	C26H45NO5S	Bile acids	Bile acids	6042-32-6	1.454	0.05267	1017.316	9.991	up
Val-Gly	C7H14N2O3	Amino acid and Its metabolites	Small Peptide	686-43-1	1.557	0.00106	0.253	-1.981	down
Val-Thr	C9H18N2O4	Amino acid and Its metabolites	Small Peptide	72636-02-3	1.143	0.00011	0.188	-2.407	down
Val-Val	C10H20N2O3	Amino acid and Its metabolites	Small Peptide	3918-94-3	1.754	0.00315	12341.979	13.591	up
Xanthine	C5H4N4O2	Nucleotide And Its metabolites	Nucleotide And Its metabolites	69-89-6	1.733	0.22750	726295.648	19.470	up

Xanthosine	C10H12N4O6	Nucleotide And Its metabolites	Nucleotide And Its metabolites	146-80-5	1.037	0.09363	2.371	1.245	up
cyclo(gly-pro)	C7H10N2O2	Amino acid and Its metabolites	Small Peptide	3705-27-9	1.752	0.00247	11398.539	13.477	up
gamma-Glu-Ala	C8H14N2O5	Amino acid and Its metabolites	Small Peptide	5875-41-2	1.273	0.00348	0.000	-14.730	down
